# The Structure, Morphology, and Complex Permittivity of Epoxy Nanodielectrics with In Situ Synthesized Surface-Functionalized SiO_2_

**DOI:** 10.3390/polym13091469

**Published:** 2021-05-01

**Authors:** Mohammed Mostafa Adnan, Inger-Emma Nylund, Aleksander Jaworski, Sverre Hvidsten, Marit-Helen Glomm Ese, Julia Glaum, Mari-Ann Einarsrud

**Affiliations:** 1 Department of Materials Science and Engineering, NTNU Norwegian University of Science and Technology, 7491 Trondheim, Norway; mohammed.m.adnan@ntnu.no (M.M.A.); inger-emma.nylund@ntnu.no (I.-E.N.); julia.glaum@ntnu.no (J.G.); 2 Department of Materials and Environmental Chemistry, Stockholm University, 106 91 Stockholm, Sweden; aleksander.jaworski@mmk.su.se; 3 SINTEF Energy Research AS, 7034 Trondheim, Norway; Sverre.Hvidsten@sintef.no (S.H.); marit-helen.ese@sintef.no (M.-H.G.E.)

**Keywords:** nanocomposites, electrical insulation, dielectric properties, filler dispersion, in situ synthesis, sol–gel

## Abstract

Epoxy nanocomposites have demonstrated promising properties for high-voltage insulation applications. An in situ approach to the synthesis of epoxy-SiO_2_ nanocomposites was employed, where surface-functionalized SiO_2_ (up to 5 wt.%) is synthesized directly in the epoxy. The dispersion of SiO_2_ was found to be affected by both the pH and the coupling agent used in the synthesis. Hierarchical clusters of SiO_2_ (10–60 nm) formed with free-space lengths of 53–105 nm (increasing with pH or SiO_2_ content), exhibiting both mass and surface-fractal structures. Reducing the amount of coupling agent resulted in an increase in the cluster size (~110 nm) and the free-space length (205 nm). At room temperature, nanocomposites prepared at pH 7 exhibited up to a 4% increase in the real relative permittivity with increasing SiO_2_ content, whereas those prepared at pH 11 showed up to a 5% decrease with increasing SiO_2_ content. Above the glass transition, all the materials exhibited low-frequency dispersion effect resulting in electrode polarization, which was amplified in the nanocomposites. Improvements in the dielectric properties were found to be not only dependent on the state of dispersion, but also the structure and morphology of the inorganic nanoparticles.

## 1. Introduction

Nanodielectrics, which are often defined as polymer composites containing filler particles smaller than 100 nm, have attracted interest for applications in high-voltage insulation due to their potentially higher dielectric breakdown strengths and lower complex permittivities, compared to unfilled polymers [1,2,3]. These benefits have been primarily attributed to the inclusion of nano-sized particles and the subsequent increase in interfacial regions between the organic matrix and inorganic filler [4]. However, the improvements in dielectric properties of nanocomposites is inconsistent across multiple studies [1], which is most likely due to variations in material processing and the resulting dispersion of the incorporated nanoparticles [5]. Dispersion of inorganic nanoparticles in an organic matrix is a challenging aspect of the processing, as the particles tend to agglomerate to reduce the high surface energy. This surface energy can be reduced by improving the compatibility between the inorganic and organic components, which is usually done using coupling agents, ligands, or other types of surface modifiers [6]. Therefore, understanding the interactions in the interfacial regions is important for explaining the properties of the nanocomposite materials. 

Among such materials, epoxy resin-based composites are commonly used in power equipment (cast-resin transformers, rotating machines, switchgear insulators, bushings, terminations, etc.) as high-voltage insulation, as well as other electrical applications, such as printed circuit boards [1,2,7]. Epoxy possesses good chemical resistance, high thermal stability, high tensile strength and toughness [6], making it suitable for such applications where the insulation is subjected not only to electrical stresses, but also thermal and mechanical stress from equipment operations [8]. 

Dielectric materials require a high dielectric strength as well as low power loss for applications in high-voltage apparatus. Since the power loss is proportional to both the real and imaginary parts (dielectric loss tangent) of the permittivity, the complex permittivity should also be low. Nelson and Fothergill [9] were among the first to demonstrate improvements in the dielectric properties of epoxy with the incorporation of inorganic nanoparticles. Since then, several others have reported similar results with a variety of different nanoparticles. Singha and Thomas presented reduced permittivities in epoxy-TiO_2_ nanocomposites. Kochetov et al. [10] demonstrated similar improvements in epoxy nanocomposites containing AlN, MgO, or Al_2_O_3_. Virtanen et al. [11], Bell et al. [12], and Yeung and Vaughan [13] all reported improved short term dielectric breakdown strengths in epoxy-SiO_2_ nanocomposites with various organic surface modifications. However, to the best of our knowledge, most studies investigating the dielectric properties of epoxy-based nanocomposites have used an ex situ processing route, where pre-synthesized nanoparticles (which may be surface-functionalized) are added to the epoxy resin and dispersed by physical methods (high shear mixing, blending, or sonication). Control of the state of dispersion of the nanoparticles, which is believed to be critical to the dielectric properties of the nanocomposites [5], is more difficult in such an approach, which often leads to agglomeration and degradation of the dielectric properties [1,5].

Alternative techniques can be employed in the preparation of the nanocomposites to improve the state of dispersion. In situ sol–gel processes can be applied to synthesize nanoparticles directly in the polymer matrix [14]. The sol–gel method is typically used for the synthesis of inorganic oxides (e.g., SiO_2_, TiO_2_, Al_2_O_3_) from the hydrolysis and polycondensation of metal alkoxide precursors [15]. Parameters such as the pH, length of the alkyl chains, solvent, and chelating agents, can be used to adjust the size and morphology of the inorganic network formed by controlling the hydrolysis and condensation reactions. The use of silane coupling agents (SCA) in the sol–gel process can result in the formation of Class II hybrid materials, where strong chemical bonds are present between the inorganic and organic components, resulting in an improved compatibility between the hydrophobic polymer and hydrophilic filler [16]. 

Matějka et al. [17,18] prepared epoxy-SiO_2_ nanocomposites using both one- and two-stage sol–gel processes resulting in branched polysiloxane clusters and aggregated clusters, respectively. The use of both acid- and base-catalysis resulted in different SiO_2_ morphologies. Nazir et al. [19] and Afzal et al. [20] adapted the former methods in a two-step chronological sol–gel process with acid catalysis and the incorporation of SCA. The use of SCA led to improved dispersion of the in situ formed SiO_2_. Donato et al. [21,22] prepared similar epoxy-SiO_2_ nanocomposites, but with the aid of ionic liquids instead of SCA for improving the dispersion further.

Although many such variations of the sol–gel method have been used in several studies to prepare epoxy-SiO_2_ nanocomposites, none of them, to the best of our knowledge, have investigated the dielectric properties of the materials prepared in this way. Additionally, very few works have employed the use of quantitative methods for characterizing the state of dispersion, which is a useful tool for limiting subjective interpretations of the degree of dispersion [5]. Various statistical methods, such as the interparticle distance, quadrat-based particle counting, and free-space length have been proposed, each with their own advantages and disadvantages [5,23]. 

Therefore, the purpose of this study was to investigate if epoxy nanocomposites fabricated using the in situ route presents reduced permittivity and dielectric loss, compared to nanocomposites fabricated using more conventional ex situ methods as reported in the literature. In the present work, epoxy-SiO_2_ nanocomposites were prepared based on a sol–gel route described in our previous study [24], using different synthesis conditions (pH, SCA amount, and type). The morphology and structure of the inorganic network and the resulting complex permittivity of the nanocomposites were characterized. Additionally, the state of dispersion was quantitatively described by applying the free-space length method. An important aspect of the work is to increase the understanding of the structure-property relations in these materials. This will allow one to tune the properties as required, such as decreasing the dielectric loss or real permittivity, or increasing the dielectric strength for high-voltage insulation materials, by tailoring the structure via alteration of the synthesis conditions.

## 2. Materials and Methods

The epoxy resin was prepared using diglycidyl ether of bisphenol-A (DGEBA) as the epoxy monomer and poly(propylene glycol) bis(2-aminopropyl ether) as the curing agent. Tetraethyl orthosilicate (TEOS) was used as the precursor for the SiO_2_ in the sol–gel reaction. 3-(aminopropyl) triethoxysilane (APTES) and 3-(glycidyloxypropyl) trimethoxy-silane (GPTMS) were the silane coupling agents used to create the interfacial link between the in situ formed SiO_2_ nanoparticles and the epoxy chains. Ammonia solution (35%) and HCl (36%) was used to alter the pH of the distilled water used for the hydrolysis and condensation of tetraethyl orthosilicate. All chemicals had a purity of >98% and were obtained from Merck Life Science AS, Oslo, Norway.

Pure epoxy samples were prepared by mixing stoichiometric amounts of DGEBA and the curing agent in a PET beaker at room temperature. This was performed under vacuum to remove all the air bubbles present in the mixture. The resin mixture was injected into a stainless-steel mold (for disc-shaped samples with 1 mm thickness and 40 mm diameter) under vacuum. The mold was placed in a pressure chamber at 100 °C for 5 h with 10 bars of N_2_ pressurization to collapse any remaining air bubbles. Leftover resin was casted in Teflon cups without pressurization to prepare bulk samples (over 5 mm in thickness).

**Scheme 1:**
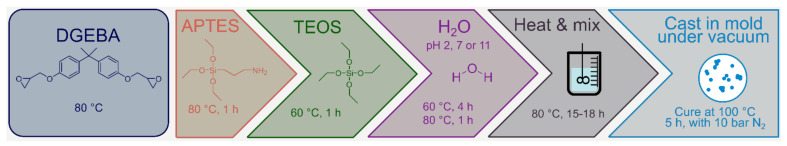
An outline of the sol–gel procedure used in the in situ synthesis of SiO_2_ functionalized with APTES in epoxy.

The composites were prepared using the procedure outlined in our previous work [24] and is illustrated in Scheme 1. DGEBA was heated to 80 °C in a round bottom flask mounted with a reflux condenser. The silane coupling agent, either APTES or GPTMS, was mixed with the DGEBA for 1 h (at 80 °C) using a magnetic stirrer (mass ratio of SCA:DGEBA was equal to either 1:10 or 1:30), followed by mixing the required amount of TEOS for 1 h (60 °C). Distilled water was then added to initiate the TEOS hydrolysis, and the mixture was stirred for a further 4 h at 60 °C, then 1 h at 80 °C. Composites were prepared using water with pH 2, pH 7 (neutral) and pH 11. Afterwards, the reaction mixture was poured into a beaker and stirred overnight (15–18 h) at 80 °C to remove alcohol byproducts. Finally, the curing agent was added to the mixture and the samples were casted using the aforementioned procedure. Table 1 shows an overview of the compositions of the various samples investigated. 

Fourier transform infrared (FTIR) spectroscopy was performed using a Bruker Vertex 80v spectrophotometer with an attenuated total reflectance (ATR) diamond cell (Bruker Corporation, Billerica, Massachusetts, USA). Each sample was scanned 32 times at a resolution of 1 cm^−1^. Small angle X-ray scattering (SAXS) measurements were performed with a Bruker NanoSTAR instrument with a Cu micro-source (Bruker Corporation, Billerica, Massachusetts, USA), operating at 50 kV and 600 μA (scattering vector (*q*) range of 0.009–0.3 Å^−1^). The data from the SAXS was analyzed using software SasView 5.0.1 (http://www.sasview.org), and fitted to the unified exponential/power-law model [25]. The Porod exponents, also known as fractal dimension (*D*), for each structural level in the model were obtained from the slopes of the linear regions after each feature. From the fits, the radii of gyration (R_g_) for each structural level were obtained, from which the inorganic domain size was calculated (assuming spherical domains), using:

(1) $$2\sqrt{5/3}\left(R_{g}\right).$$

The correlation length (ζ) between inorganic structures or domains was estimated using [26]:

(2) $$\zeta =\frac{2\pi }{q},$$

where *q* is the scattering vector for the peak or feature.

Transmission electron microscopy (TEM) was performed using a JEOL JEM 2100F (JEOL Ltd., Tokyo, Japan), at 200 kV accelerating voltage, on 50–100 nm slices of the samples cut using an ultramicrotome. Energy-dispersive X-ray spectroscopy (EDS) was performed using an Oxford X-Max 80 SDD detector (Oxford Instruments, Abingdon, UK) attached to the TEM instrument. Particle cluster sizes were determined visually from the TEM images. The quantitative analysis of the dispersion of the nanoparticles was performed using MATLAB 202a (Mathworks, Portola Valley, CA, USA) and the code and methodology provided by Khare and Burris [23]. The TEM images were processed into the binary images required for the analysis using ImageJ 1.52a. 

A Netzsch DSC 214 Polyma (NETZSCH-Gerätebau GmbH, Selb, Germany) was used to perform differential scanning calorimetry (DSC) between 0 and 200 °C in N_2_ atmosphere (4 cycles, 10 °C/min heating and cooling rates, 40 mL/min gas flow). The glass transition temperature (T_g_) was obtained from the local maxima during the increase in the heat capacity.

^1^H→^29^Si cross-polarization magic-angle spinning (CPMAS) NMR spectra were collected on a Bruker Avance-III NMR spectrometer (Bruker Corporation, Billerica, MA, USA) at a magnetic field strength of 14.1 T (Larmor frequencies of 600.1 and 119.2 MHz for ^1^H and ^29^Si, respectively) using 7.0 mm zirconia rotors at a MAS rate of 5.00 kHz. Acquisitions involved proton 90° excitation pulse of 4 μs and matched spin-lock fields of ν_H_ = 60 kHz and ν_C_ = 40 kHz. Contact time of 5 ms was used and SPINAL-64 proton decoupling at 60 kHz. Between 16384 and 28672 signal transients with 4 s relaxation delays were collected per sample. Chemical shifts were referenced with respect to neat tetramethylsilane (TMS). Peak deconvolution was performed using Origin 2018b (OriginLab Corporation, Northhampton, MA, USA), and the degrees of condensation for Q and T species were calculated from the peak areas using Equations (3) and (4), respectively:

(3) $${\left[\alpha_{Si}\right]}_{Q}=\frac{\sum iQ_{i}}{4},$$

(4) $${\left[\alpha_{Si}\right]}_{T}=\frac{\sum iT_{i}}{3}.$$

Broadband dielectric spectroscopy was conducted using a Novocontrol Spectrometer with an Alpha Beta dielectric analyzer (Montabaur, Germany). A BDS1200 sample cell with 1 V/mm electric field was used to measure the samples’ dielectric response between 10^−2^ and 10^6^ Hz over 5 °C intervals from 25 to 120 °C, and 20 °C intervals from 140 to 200 °C.

## 3. Results

### 3.1. Dispersion and Morphology of the In Situ Synthesized Nanoparticles

**Figure 1:**
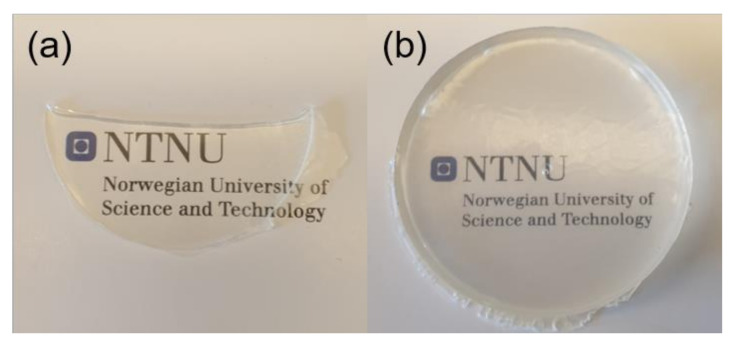
Representative images of transparent epoxy-SiO_2_ nanocomposites (5 wt.% SiO_2_ with APTES at pH 11) after casting. (**a**) A 1 mm thick sample casted under N_2_ pressurization. (**b**) A thick sample (>5 mm) casted without N_2_ pressurization.

The primary motivation for the use of the in situ approach in the synthesis of the nanocomposites was to ensure that the nanoparticles formed were well dispersed with limited agglomeration. The composites prepared with APTES as the SCA were transparent for the composites casted both with and without N_2_ pressurization, as shown in Figure 1, indicating high degree of dispersion.

**Figure 2:**
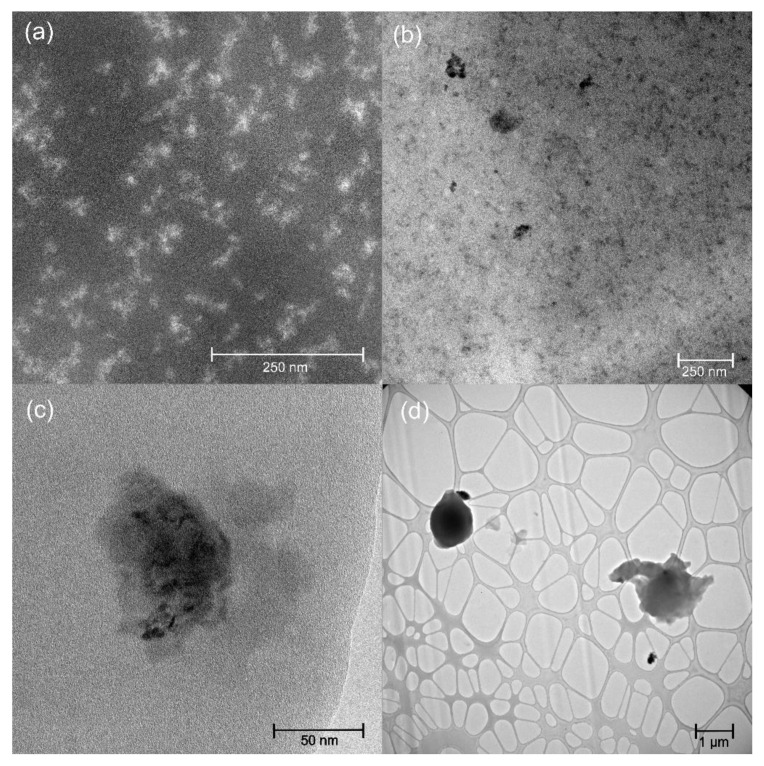
(**a**) Representative HAADF-STEM image of epoxy nanocomposite (5 wt.% SiO_2_) prepared using pH 7 and APTES. (**b**) Representative TEM image of epoxy nanocomposite (5 wt.% SiO_2_) prepared using pH 11 and APTES. (**c**) A single SiO_2_ agglomerate consisting of smaller particles in the composite prepared at pH 11 (5 wt.% SiO_2_). (**d**) Epoxy nanocomposite (5 wt.% SiO_2_) prepared using GPTMS, exhibiting large SiO_2_ agglomerates.

The scarcity of SiO_2_ agglomerates, defined in this work as particle clusters larger than 100 nm, was confirmed by TEM. Figure 2 shows representative bright field TEM and high-angle annular dark field scanning TEM (HAADF-STEM) images of the nanocomposites (all with 5 wt.% SiO_2_) prepared with APTES at pH 7 and 11, and with GPTMS at pH 2. The SiO_2_ particles form randomly dispersed clusters. For the composites prepared at pH 11 several large agglomerates (100–150 nm) consisting of multiple smaller particles (Figure 2c) were formed, compared to composites prepared using pH 2 and 7 where fewer or almost no such agglomerates were present. HAADF-STEM was used to image the smaller clusters in the nanocomposites as it provided better contrast (Figure 2a) and was also used to verify that the particle composition was SiO_2_ using energy-dispersive X-ray spectroscopy (EDS, see Figure S1). The samples prepared with GPTMS, however, showed poor dispersion of the SiO_2_, forming agglomerates in the micron range as shown in Figure 2d.

**Figure 3:**
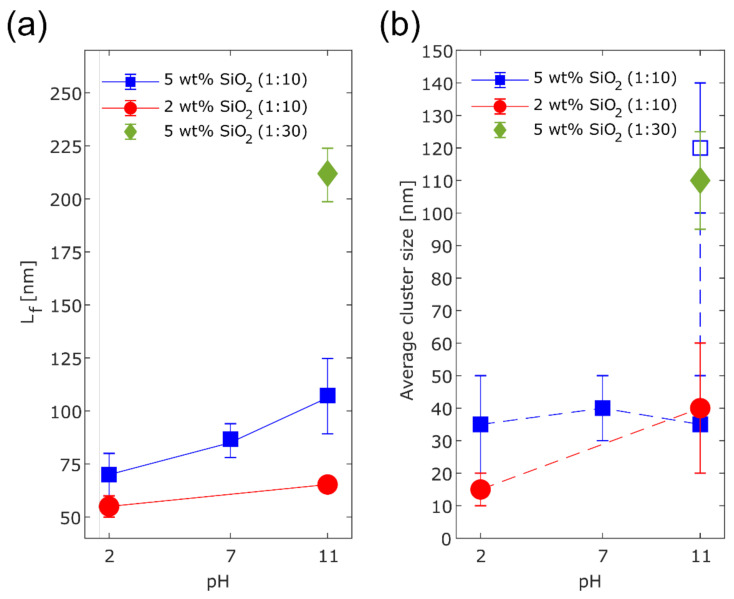
Changes in the dispersion quality with variations in pH, filler content, and amount of SCA (shown as the ratio of APTES:DGEBA in parentheses in the legend) used in the synthesis. The (**a**) L_f_ and (**b**) average cluster sizes are shown for composites prepared with 2 and 5 wt.% SiO_2_ (● and ■ respectively) and 1:10 of APTES:DGEBA at different pH. The ◆ indicates the composite prepared at pH 11 with 1:30 APTES:DGEBA and 5 wt.% SiO_2_. In (**b**), the ☐ indicates the average size of the agglomerates observed in the composite with 5 wt.% SiO_2_ (1:10 APTES:DGEBA, pH 11).

Figure 3 shows the changes in free-space length (L_f_) with variations in filler content and pH during synthesis. The calculation of L_f_ from the TEM images is described in the Supplementary Material (Figures S1 and S2, and Table S1). An increase in pH (for a given filler and SCA content) resulted in an increase in L_f_. An increase in the filler content also had the same effect. The cluster sizes were affected by the changes in pH and filler content as well. At pH 2, the clusters increased in average size with the increasing amount of SiO_2_. At pH 11, increasing amount of SiO_2_ led to a partly bimodal distribution of particle clusters, which were smaller on average than at lower SiO_2_ contents, and agglomerates of 100–150 nm, which were not as frequent or completely absent at lower SiO_2_ contents. A reduction in the amount of SCA, however, resulted in the most noticeable difference in the dispersion quality, with a doubling in the cluster sizes as well as the L_f_. 

**Figure 4:**
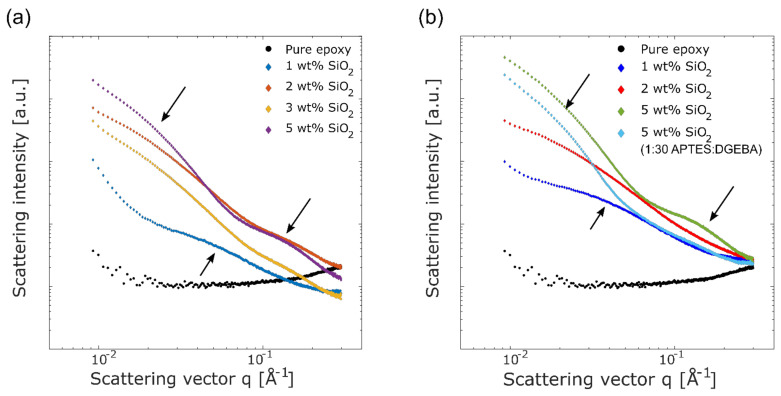
SAXS profiles for pure epoxy and epoxy-SiO_2_ nanocomposites with APTES synthesized with (**a**) pH 7 and (**b**) pH 11. The sample prepared with an APTES:DGEBA ratio of 1:30 at pH 11 is also included in (**b**). The features in the scattering pattern for the nanocomposites (shown by the arrows) indicate a hierarchical structure in the SiO_2_.

The SAXS profiles of the nanocomposites prepared at pH 7 and 11 presented in Figure 4 show increased scattering from the epoxy-SiO_2_ nanocomposites compared to pure epoxy. The emergence of broad features (often referred to as Guinier knees) in the scattering profile are indicated by the arrows. These knee-like features become more prominent with increasing SiO_2_ content and appear in the *q* range 0.07–0.24 Å^−1^ and 0.01–0.03 Å^−1^ for samples with 2 and 5 wt.% SiO_2_ at pH 7. For the sample with 1 wt.% SiO_2_ at pH 7, it appears that only one broad feature is present between 0.024 and 0.15 Å^−1^. For the nanocomposites prepared at pH 11, those with 1 and 2 wt.% of SiO_2_ also show a single broad feature between 0.02 and 0.15 Å^−1^, and the “peak” for the feature at low *q* for the 5 wt.% SiO_2_ is below the measured *q* range (<0.009 Å^−1^). The 5 wt.% SiO_2_ sample prepared with reduced APTES (1:30 of APTES:DGEBA) at pH 11 does not exhibit the second feature at higher *q*, and a more linear region of scattering is observed. In all nanocomposites, the scattering appears to increase further at *q* lower than the measured range.

The presence of multiple features indicates a hierarchical structure of the SiO_2_, which is described by the unified exponential/power-law model by Beaucage [25]. The model describes complex morphology over wide *q* ranges using structural levels—a structural level in scattering is reflected by a knee and a linear region on a log-log plot of scattering, representing Guinier’s law and a structurally limited power law, respectively [27]. A fit of this model with two structural levels was applied to the data where two broad features were observed. Table 2 shows the fractal dimension (*D*) (measured from the slopes of the linear regions after each feature) and the calculated radii of gyration (R_g_) and structure sizes (d) for the inorganic domains (obtained from the fits to the unified exponential/power-law model). The sizes of the SiO_2_ domains in the second structural level increased with increasing SiO_2_ content for nanocomposites prepared at pH 7. At the primary level, the changes were inconsistent. A similar comparison could not be made for the samples prepared at pH 11, since no suitable fits were obtained for the nanocomposites with 1 and 2 wt.% SiO_2_ at pH 11. 

The fractal dimension, which describes the power-law dependence of the scattering intensity [28], increased in all the samples with increasing SiO_2_ content at both the primary and secondary structural levels. At the primary level, 1 < *D* < 2, indicating a mass-fractal structure—in other words, the SiO_2_ clusters (8–10 nm) consist of coiled polymeric chains (with Si-O-Si links). At the secondary level, D > 2 for all samples, and increased with increasing SiO_2_ content. In nanocomposites containing 5 wt.% SiO_2_, 3 < D < 4. This indicates the formation of increasingly interconnected polymer chains forming a clustered network, and eventually the formation of particles with a rough surface (a surface-fractal structure).

### 3.2. Structure of the Inorganic Components

**Figure 5:**
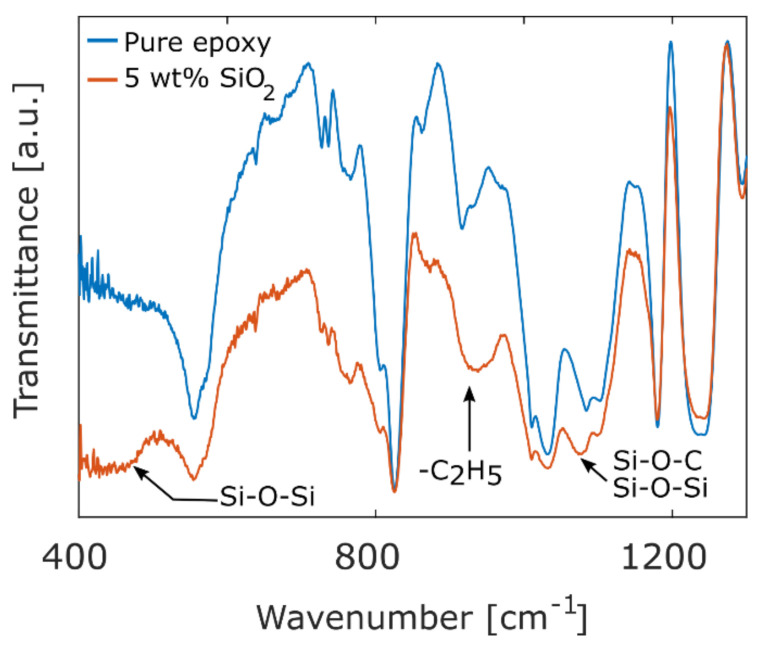
FTIR spectra of pure epoxy and epoxy-SiO_2_ nanocomposite with 5 wt.% SiO_2_ at pH 7. The relevant differences in the spectra are marked.

FTIR and ^29^Si solid-state NMR provided further information on the bonds formed in the nanocomposites. Figure 5 shows the IR spectra of pure epoxy and the epoxy-SiO_2_ nanocomposite with 5 wt.% SiO_2_ (pH 7), both normalized for the band at 1500 cm^−1^ (not shown in the figure). The features specific for the nanocomposite spectrum are the presence of the O-Si-O rocking band around 450 cm^−1^, the band at 940–970 cm^−1^ for the ethyl (-C_2_H_5_) groups from unreacted precursors and coupling agents, and a broader band at 1080–1100 cm^−1^ assigned to the Si-O-Si and Si-O-C stretching [29]. A more detailed description of the progress of the in situ sol–gel reactions and formation of the inorganic domains is provided in our previous work [24]. 

**Figure 6:**
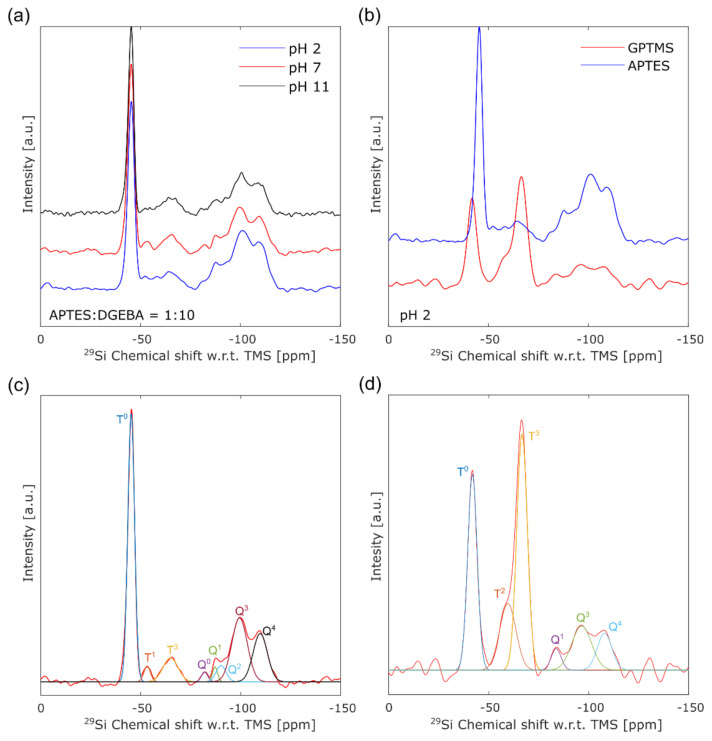
^29^Si NMR (CP-MAS) spectra of epoxy-SiO_2_ nanocomposites. (**a**) Nanocomposites prepared with APTES (APTES:DGEBA = 1:10) at different pH. (**b**) Comparison of nanocomposites prepared with APTES and with GPTMS (both at pH 2). (**c**) Deconvolution of the peaks in the spectra for nanocomposites prepared with APTES. (**d**) Deconvolution of the peaks for nanocomposites prepared with GPTMS.

^29^Si NMR was used to ascertain the degree of condensation of the Si-O network. Figure 6 shows the NMR spectra of the nanocomposites prepared at different pH and using different SCAs, along with the deconvolution of the peaks. Each peak corresponds to either a T^x^ or Q^y^ signal (0 ≤ x ≤ 3 and 0 ≤ y ≤ 4), where *x* and *y* indicate the number of alkyl or -OH groups that have been replaced by an -O-Si bond on a central Si atom in the SCA or TEOS, respectively. The Q^3^ and Q^4^ signals are prominent, while the Q^0^, Q^1^, and Q^2^ signals are much weaker. For the APTES, the T^0^ peak is the most prominent, with weaker T^1^ and T^3^ peaks. For the nanocomposite containing GPTMS, however, the NMR spectra shows stronger T^2^ and T^3^ peaks and a weaker T^0^ peak, accompanied with the presence of Q^1^, Q^3^, and Q^4^ peaks. The degree of condensation of Q and T species, [α_Si_]_Q_ and [α_Si_]_T_, were calculated to be 0.77 and 0.17, respectively, for nanocomposites prepared with APTES. For the nanocomposites prepared with GPTMS, [α_Si_]_Q_ and [α_Si_]_T_ were calculated to be 0.77 and 0.59, respectively. The fractions of the structural units Q_i_ and T_i_ were obtained from the area under the peaks. No significant differences were observed between samples prepared at different pH (for a given SCA).

**Figure 7:**
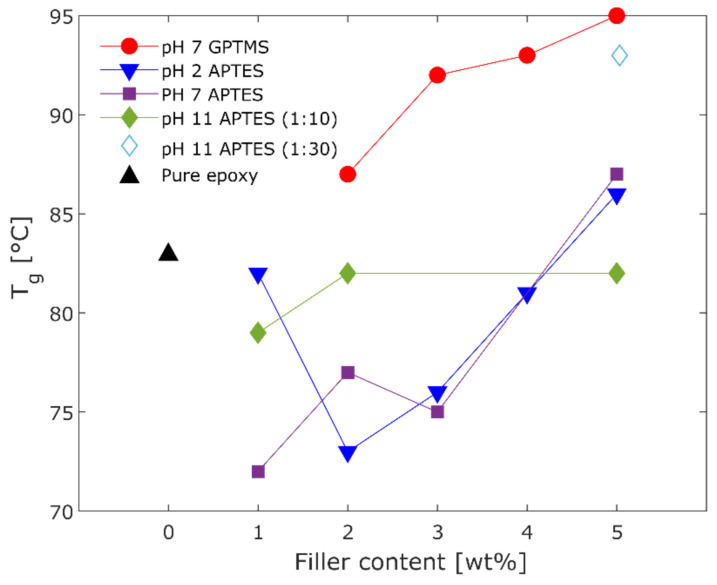
Changes in the glass transition for the epoxy-SiO_2_ nanocomposites with varying filler content prepared using APTES (with APTES:DGEBA of 1:10 and 1:30) and GPTMS with different pH conditions, compared to that of pure epoxy (0 wt.% filler content). The error in the measurements is ±2 °C. The data for the pH 2 APTES samples were taken from [24].

Figure 7 displays the glass transition temperatures (T_g_) for the various nanocomposites compared to epoxy. In the composites prepared with APTES, the initial addition of SiO_2_ resulted in a decrease in T_g_ from that of pure epoxy (83 °C). In the composites prepared at pH 2, T_g_ continues to decrease until the SiO_2_ content is above 2 wt.%, after which T_g_ increases again with further increases in SiO_2_ content. In the composites prepared at pH 7, the drop in T_g_ is much more drastic at 1 wt.% SiO_2_, but it again increases rapidly with increasing SiO_2_ content, and exceeds the T_g_ of pure epoxy. The changes in T_g_ for the composites prepared at pH 11 are comparatively less drastic, and even at 5 wt.% SiO_2_ the T_g_ is less than that of pure epoxy. However, the sample with 5 wt.% SiO_2_ prepared with less APTES (APTES:DGEBA of 1:30) exhibited quite a high T_g_ (93 °C). The nanocomposites prepared with GPTMS on the other hand show the opposite behavior, and T_g_ is seen to increase steadily with SiO_2_ content and exhibits the highest T_g_ of all the composites (up to 95 °C).

### 3.3. Complex Permittivity of the Nanocomposites

**Figure 8:**
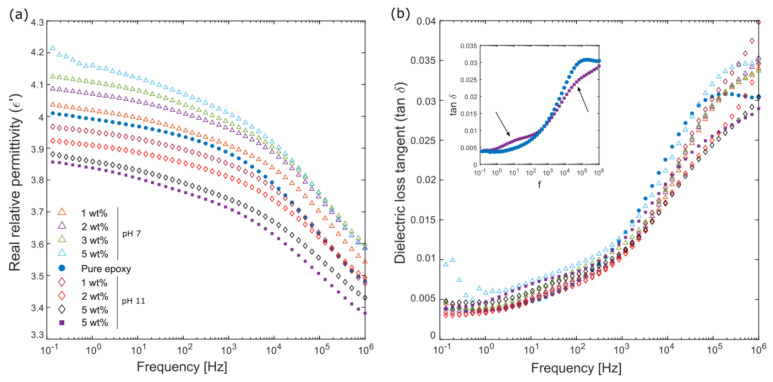
The complex permittivity of pure epoxy and epoxy nanocomposites with varying amounts of SiO_2_ at room temperature. △ indicates samples prepared with pH 7 and ◇ indicates samples prepared with pH 11 (both with 1:10 of APTES:DGEBA), while ■ indicates the sample prepared with pH 11 and 1:30 of APTES:DGEBA. (**a**) The real relative permittivity (ε’). (**b**) The dielectric loss tangent (tan δ). The inset in (**b**) shows the same data for pure epoxy and epoxy-SiO_2_ nanocomposite, with the arrows highlighting the different features in tan δ. The legend in (**a**) is common for both figures.

The room temperature complex permittivities of pure epoxy and the in situ nanocomposites prepared with APTES are shown in Figure 8. The real part of the relative permittivity exhibited a small increase with increasing SiO_2_ content for the nanocomposites prepared at pH 7. On the contrary, for the nanocomposites prepared at pH 11, the permittivity decreased with increasing SiO_2_ content. Additionally, the sample with 5 wt.% SiO_2_ prepared at pH 11 and with a lower amount of APTES (APTES:DGEBA = 1:30) exhibited the lowest real relative permittivity. 

The dielectric loss tangent (tan δ) for pure epoxy displays a peak at 1∙10^5^–2∙10^5^ Hz, which is henceforth called the β-relaxation peak. The nanocomposites exhibit small differences in the loss tangent, with the most noticeable being the emergence of a new feature with a much smaller peak height, between 1 and 10^3^ Hz (indicated by the left arrow in the inset in Figure 8b). Another feature is observed at frequencies above the β-relaxation peak (10^5^–10^6^ Hz), and the β-relaxation appears as a shoulder on this new relaxation, which has its peak beyond the measurement range. The nanocomposites show small increases in the dielectric loss with increasing SiO_2_ content for the new relaxation at 1–10^3^ Hz. Near the β-relaxation peak, the dielectric loss is decreased for nanocomposites at frequencies below 10^5^ Hz, but at higher frequencies the dielectric loss increased (due to the new relaxation that had emerged). An exception to this behavior was observed in nanocomposites prepared at pH 11, where the dielectric loss at high frequencies decreased significantly compared to pure epoxy (as indicated by the right arrow in the inset in Figure 8b), especially for higher SiO_2_ contents. The peak for tan δ is lower, but it remains a shoulder on the new relaxation. 

**Figure 9:**
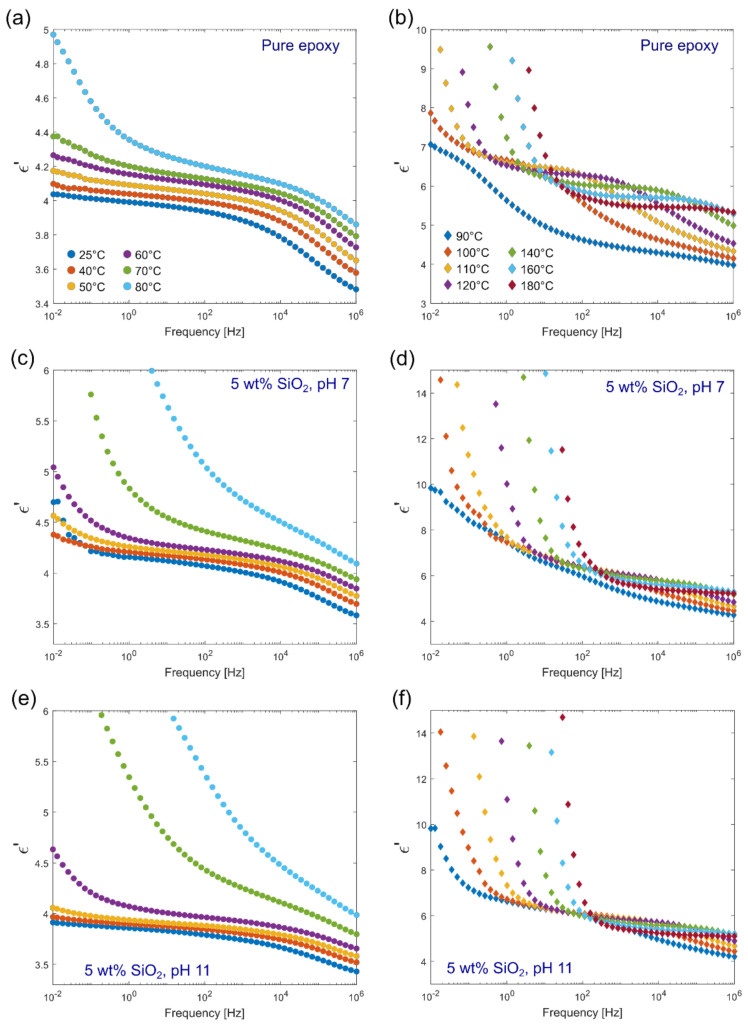
The real relative permittivities (ε’) for (**a**,**b**) pure epoxy, and epoxy nanocomposites prepared with 5 wt.% SiO_2_ at (**c**,**d**) pH 7, and (**e**,**f**) pH 11. The plots on the left (**a**,**c**,**e**) show the permittivities below 80 °C, while the plots on the right (**b**,**d**,**f**) show the permittivities above 80 °C.

**Figure 10:**
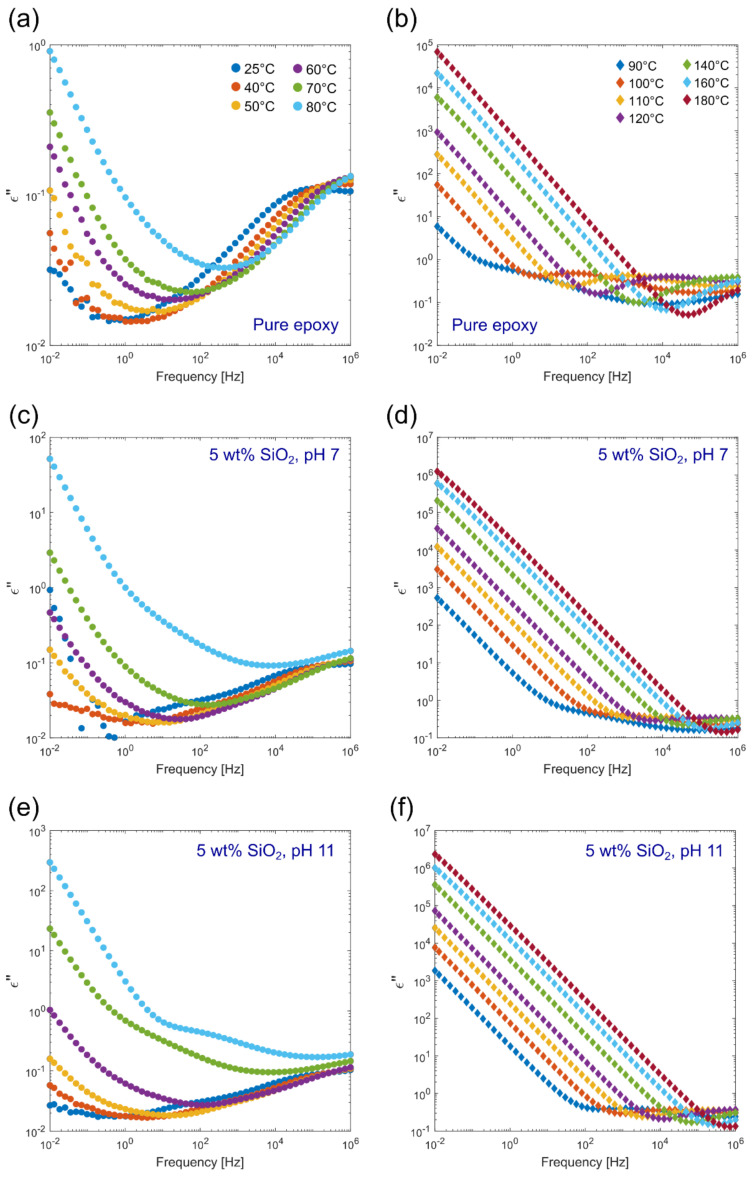
The imaginary permittivities (ε’’) for (**a**,**b**) pure epoxy, and epoxy nanocomposites prepared with 5 wt.% SiO_2_ at (**c**,**d**) pH 7, and (**e**,**f**) pH 11. The plots on the left (**a**,**c**,**e**) show the permittivities below 80 °C, while the plots on the right (**b**,**d**,**f**) show the permittivities above 80 °C.

Figure 9 displays the real relative permittivities (ε’) of pure epoxy and selected nanocomposites from 25 to 200 °C, and Figure 10 shows the corresponding imaginary permittivities (ε’’) over the same temperature range. As the temperature is increased, the permittivity increased and the β-relaxation around 10^5^ Hz shifted to higher frequencies for all the samples. This is observed more clearly from the peak positions in the imaginary permittivity. As the glass transition was approached (between 60 and 90 °C), a new relaxation shifted into the measurement range at low frequencies. This occurred at lower temperatures for the nanocomposites (60 °C) than for the pure epoxy (80 °C). Additionally, this relaxation was observed at higher frequencies as the temperature increased, which is similar to the behavior shown by the β-relaxation. Beyond T_g_, the permittivity increased exponentially at low frequencies to very high values. For the nanocomposites, this development is more severe than for the pure epoxy, starting at lower temperatures and reaching higher values (above 10^3^). At low frequencies and high temperatures, ε’’ becomes linear with a slope between −0.9 and −1. This linear region extends to higher frequencies as the temperature is increased further. Above 140 °C the slope of ε’ was approximately −1.

## 4. Discussion

### 4.1. The Effect of the SCA on the State of Dispersion

**Figure 11:**
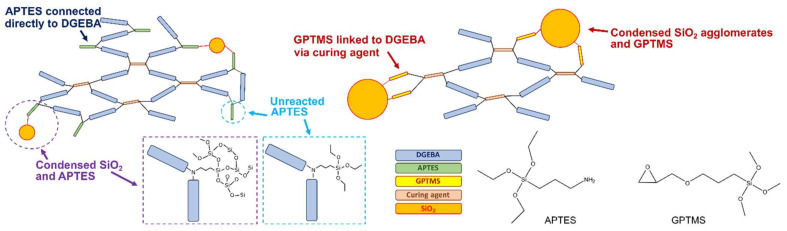
Schematic showing the structure of the two SCAs used and how they form a chemical link between the inorganic and organic components in the nanocomposites.

The reaction mechanisms for the formation of SiO_2_ are different when APTES or GPTMS is used as the SCA. This can be attributed to the structures of the SCAs and how they interact with the DGEBA, as shown schematically in Figure 11. APTES contains a -NH_2_ group, which is capable of bonding directly with DGEBA and forming cross-links [24]. GPTMS on the other hand contains epoxide groups, and, therefore, cannot bond with DGEBA directly. The connections to the epoxy chains are formed after the addition of the curing agent, which also contains -NH_2_ groups that form the cross-links between DGEBA as well as between DGEPA and GPTMS. Therefore, in the synthesis procedure followed in this work, APTES can immediately link with the DGEBA monomers when it is initially mixed, attaching to the ends of various DGEBA chains and forming multiple sites for the SiO_2_ to anchor to. GPTMS is unable to do this, as the curing agent is not added until the final stage of the synthesis. 

**Figure 12:**
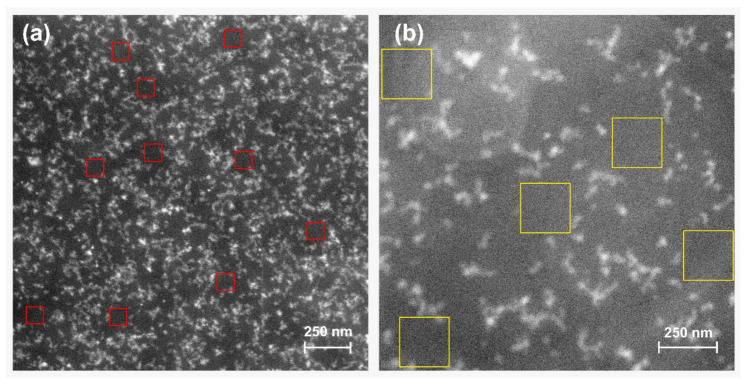
Comparison of the state of dispersion and morphology between SiO_2_ prepared at pH 11 with an APTES:DGEBA ratio of (**a**) 1:10 and (**b**) 1:30, both at filler load of 5 wt.%. The red and yellow boxes visualize the size of the free-space lengths (L_f_) in each composition (~105 and 205 nm, respectively).

The T^0^ signal from the NMR spectra (Figure 6) is observed at a chemical shift of −45 instead of the expected −40 to −43 [30,31]. Hoebbel et al. [30] reported that the T^0^ chemical shift becomes larger (more negative) with fewer -OH and more -OC_2_H_5_ groups attached to the Si in the SCA, thus indicating that some of the APTES is not completely hydrolyzed. This is also verified by the presence of Si-O-C_2_H_5_ groups as shown in the FTIR spectrum (Figure 5). It is, therefore, likely that APTES shows less self-condensation due to the anchoring to the DGEBA first. Piscitelli et al. [31,32] reported fully condensed GPTMS and SiO_2_ (from TEOS) when prepared using a similar aqueous sol–gel method at pH 6 and the use of a condensation catalyst (dibutyltindilaurate). The use of such a condensation catalyst may help in ensuring that all the SiO_2_ formed is connected to the APTES as it will be fully condensed, but also increases the chance of self-condensation of APTES. From the TEM images (Figure 2), it is seen that in all the samples prepared with APTES the SiO_2_ is randomly distributed in well-dispersed nanoparticle clusters, with only some agglomeration at pH 11. A reduction in the amount of APTES by a third affects the dispersion quality noticeably, resulting in doubling the average cluster size and free-space length (L_f_) (Figure 3). Figure 12 shows the differences in the state of dispersion when the mass ratio of APTES:DGEBA is changed (from 1:10 to 1:30). More discrete particle clusters with a larger average distance between the clusters (larger L_f_ of approximately 205 nm) form with a reduced amount of APTES. With fewer APTES molecules and fewer binding spots to the DGEBA chains, the SiO_2_ subsequently forms structures that are larger, but fewer in number (in contrast to the multitude of smaller structures) and spread much further apart.

With GPTMS, however, the SCA self-condensates alongside TEOS, as observed from the higher degree of condensation [α_Si_]_T_ for the T species in GPTMS (Figure 6b). Due to this and the inability of GPTMS to anchor to the DGEBA chains without the curing agent, the samples prepared with GPTMS form large agglomerates of SiO_2_ over 1 μm (Figure 2d).

Pre-synthesized SiO_2_ nanoparticles, typically spherical or with a defined particle shape, have been found to be difficult to disperse in epoxy in traditional ex situ methods of preparation [1]. The in situ approach used in this work can consistently prepare nanocomposites with a homogeneous dispersion of the SiO_2_ formed when APTES is used as the coupling agent, and the dispersion can be controlled by the synthesis parameters, as seen from the consistent changes in L_f_ with pH, filler content, and amount of APTES. However, unlike the pre-synthesized nanoparticles, the structures formed by SiO_2_ in this in situ sol–gel route show greater variance, with the formation of an inorganic network instead of discretely shaped particles. 

### 4.2. The Structure of the In Situ Synthesized SiO_2_

The morphology and organization of the SiO_2_ structures formed in the epoxy is quite different from when ex situ nanocomposites are prepared. From the SAXS measurements (Figure 4) it is seen that at higher SiO_2_ contents, the SiO_2_ domains have formed two structural levels in a hierarchical organization: the first level consisting of clusters consisting of polymeric chains of Si-O-Si links, formed from the hydrolysis of TEOS, exhibiting a mass-fractal structure; the second structural level consists of larger, networked clusters consisting of several of these mass-fractal chains. The exact correlation lengths between the clusters could not be determined as the “peaks” of these features are difficult to identify, but they were estimated to be approximately 4.2–6.3 nm and 25–42 nm for the primary and secondary structural levels (or inorganic domains), respectively. For samples exhibiting just a single broad feature, the correlation length was estimated to be in the range 9–15 nm. For samples with 5 wt.% SiO_2_ prepared at pH 11, the peak for the secondary structural level was not resolved within the resolution limits (as seen from the scattering profile at low *q*), so the correlation length between the inorganic domains must be larger than 70 nm. The calculated correlation lengths of 25–42 nm are much smaller than the calculated free-space lengths (L_f_) (53–120 nm, as seen in Table S1). The discrepancy highlights one of the limitations of using quantitative methods in characterizing the dispersion quality from two-dimensional images. However, such techniques are still useful for highlighting trends in the state of dispersion, and to independently corroborate the results from other measurements. In this case, from the calculated values of both the correlation lengths from SAXS and the free-space lengths from TEM images, the same effect is observed with increasing SiO_2_ content: an increase in the distance between the larger SiO_2_ domains (secondary structures), as well as a small increase in the sizes of the domains. It should also be noted that the scattering appears to be increasing at even lower *q* than measured. This suggests that there might be an additional structural level. From the TEM images in Figure 2 and Figure 12, it can be assumed that the additional structural level may be an arrangement of the secondary particle clusters to form more mass-fractal structures (spanning above 50–100 nm), or in some cases larger (>200 nm) agglomerates. Additionally, reducing the amount of APTES is also seen to alter the scattering profile (Figure 4b) where the knee-like feature at higher *q* is replaced by a more linear region with a Porod slope of −1.2. The slope of the linear region at *q* < 0.04 Å^−1^ is 3.5. The absence of an obvious knee-shaped feature at *q* > 0.05 Å^−1^ may be indicative of a lack of hierarchical organization, and instead the presence of both mass-fractal and surface-fractal structures in the same size region (resulting in an overlap in the scattering profile). 

**Figure 13:**
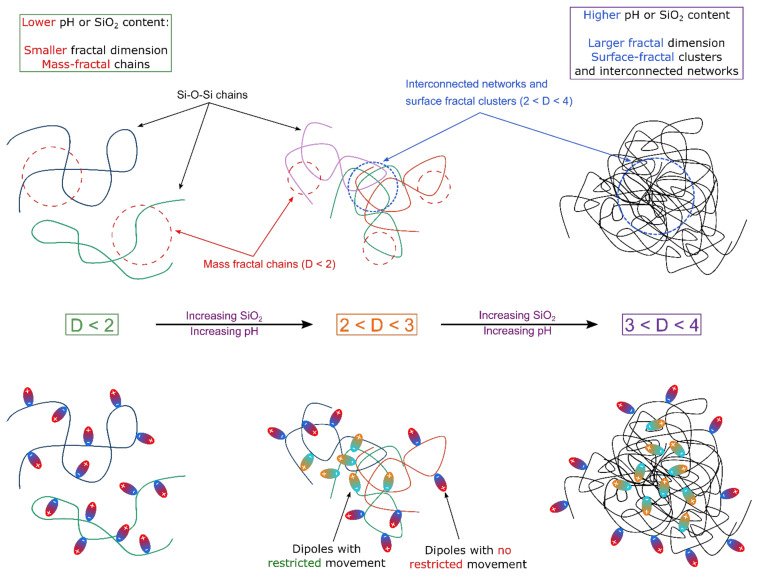
Illustration of the evolution of the SiO_2_ structures forming in situ with increasing pH and SiO_2_ content, from polymeric mass-fractal chains of Si-O-Si links to interconnected networks and rough particles with surface-fractal clusters. The resulting changes in the mobility of the chains and the freedom of the dipoles (-OH groups from hydrolysis of TEOS) to move and reorient in an electric field are shown as well.

The changes in the SiO_2_ structure with the filler content indicate a possible evolution in the growth mechanism. From the NMR spectra (Figure 6), there are very few Q^0^ groups observed in the nanocomposites, meaning that there are few unreacted TEOS monomers. This indicates that the primary growth mechanism in the initial stages is cluster-cluster, with a strong hydrolysis and slow condensation with limited monomers [15]. This mechanism results in the more open mass-fractal structures observed for low SiO_2_ contents. This is also observed from the SAXS analysis, with *D* < 3 for both the primary and secondary structural levels (Table 2). As the SiO_2_ content is increased, a larger amount of TEOS is required (meaning a larger number of monomers) and after the initial cluster-cluster reactions, a monomer-cluster mechanism steadily takes over the growth process, causing the SiO_2_ structures to become more closed and compact with more surface fractals (3 < *D* < 4) [15]. The increase in *D* for both the primary and secondary structural levels in the inorganic domains represents an increase in the compactness and cross-linking of the structures formed. Figure 13 illustrates the change in structure of the SiO_2_ from mass-fractal to surface-fractal with increasing fractal dimension. The growth mechanism is, of course, also affected by the pH of the system—classically, a lower pH would increase the rate of hydrolysis in a silica sol–gel reaction, while a higher pH would increase the rate of condensation [15]. However, this effect is less evident in the present samples as the [α_Si_]_Q_ does not change significantly with pH (as seen in Figure 6a). The effect of the pH is more prominent when looking closer at the dispersion quality—the faster condensation at higher pH leads to more compact clusters that are spaced further apart and have a higher fractal dimension, along with more agglomerates, resulting in a larger L_f_ (Figure 3). Meanwhile, the faster hydrolysis at lower pH results in fewer agglomerates, and the primary clusters are more open and spaced closer to one another, leading to a smaller L_f_ and a lower fractal dimension.

Silica derived from alkoxides via a sol–gel process is known for generally possessing less dense non-colloidal particles with fractal arrangements [15]. Several studies have demonstrated fractal structures for SiO_2_. Lysenkov et al. [33] performed a similar analysis of their corresponding SAXS measurements of in situ prepared SiO_2_ in epoxy using the exponential model. In their work, however, the clusters and agglomerates formed at higher SiO_2_ contents were much larger, and displayed varying hierarchies (e.g., mass to surface to mass fractals, or mass to mass to surface fractals), while in the present work only the evolution from a mass-fractal to a surface-fractal structure is observed with increasing SiO_2_ content. These differences in the morphology might be attributed to the lack of any SCA or other surface modifiers in Lysenkov’s study, as well as differences in the synthesis procedure. Ponyrko et al. [34] showed changes in the scattering profile depending on whether an aqueous or nonaqueous sol–gel method was employed, resulting either in compact aggregates of SiO_2_ or more open and branched aggregates, respectively. Perchacz et al. [35] similarly reported the formation of either mass-fractal or surface-fractal SiO_2_ in epoxy depending on the type of catalyst used (amine or tin-based, respectively) during the in situ synthesis, although a hierarchical structure was not evident from the flatter features in the corresponding SAXS profiles shown in their work.

### 4.3. The Effect of the SiO_2_ on the Complex Permittivity at Room Temperature

The presence of SiO_2_ is observed to affect the complex permittivity of epoxy differently depending on the synthesis conditions. One common feature present in all the nanocomposites is the emergence of a new dielectric relaxation that is associated with the SiO_2_ (between 10^0^ Hz and 10^3^ Hz) as it is missing in pure epoxy (Figure 8b inset). This relaxation occurs at a lower frequency than the eminent β-relaxation, which is associated with the dipoles on the O-H groups in the epoxy chains (or possibly from any unreacted amine groups in the curing agent) [36,37]. The dipoles associated with this new relaxation are therefore ‘stiffer’ than the β-relaxation and are suspected to be related to interfacial polarization effects at the surfaces of the nanoparticle clusters that are formed. An additional effect is the change in the β-relaxation. In pure epoxy the tan δ peak for the β-relaxation (around 10^5^ Hz) is asymmetrical, which indicates that there is a distribution in the relaxation times of the O-H dipoles, which is not unexpected due to the varying lengths and conformations of the cross-linked DGEBA chains. For the nanocomposites, the β-relaxation is altered—it appears as a shoulder (around 10^5^ Hz) on another relaxation with a higher tan δ peak at a higher frequency (beyond the measured range). This can indicate one of two possibilities—either the inclusion of the nanoparticles has introduced a new relaxation occurring in the same region as the β-relaxation, or it has shifted a pre-existing relaxation to a lower frequency region which now overlaps with the β-relaxation. The latter is less likely given that the only other relaxations that have been reported for epoxy at higher frequencies are associated with localized intramolecular motions involving the epoxide groups [38], of which there should be very few (if not none) upon complete curing. The other alternative, a new relaxation, could be related to the amine groups of any unreacted APTES, or the N-H dipoles in the cross-links formed with APTES (in addition to those in the cross-linked formed with the curing agent, which contribute already to the existing β-relaxation). Additionally, for the samples prepared at pH 11, the strength of the β-relaxation is diminished, as seen from the lower dielectric loss at high frequency. This would indicate that the more compact, networked SiO_2_ clusters formed at higher pH can restrict the mobility of the epoxy chains or the O-H dipoles more strongly than mass-fractal SiO_2_ chains.

Apart from the changes observed in the tan δ that are related to the relaxations, the real relative permittivity (ε’) at room temperature shows small changes with the SiO_2_ content and the pH used during synthesis. It is interesting to note that all the nanocomposites prepared at pH 11 show a small decrease in ε’, while those prepared at pH 7 show a small increase, compared to pure epoxy. This is likely related to the differences in structure and morphology in the SiO_2_ domains that were discussed earlier—Figure 13 shows also the subsequent effects on the dipoles present in SiO_2_ with the evolving structure. The presence of mass-fractal-like polymeric chains of SiO_2_ with a plasticizing effect on the epoxy has been reported previously [24,32], and may explain the increase in ε’ as well—the dipoles in these loose chains (such as O-H groups from hydrolyzed TEOS or surface hydroxyls in SiO_2_) can more easily and freely reorient themselves with the electric field. However, with more condensed, compact SiO_2_ structures, the dipoles are unable to keep up with the electric field as they are more restricted in such clusters—further, the rigidity of these clusters may also impede the motion of the O-H dipoles on the epoxy chains instead, thereby decreasing ε’ and decreasing the strength of the β-relaxation (tan δ above 10^3^ Hz). However, due to the new relaxation introduced by the SiO_2_, the dielectric losses are higher than that of pure epoxy between 1 and 100 Hz. Interestingly, the lowest real permittivity and dielectric loss was observed in the sample with less APTES at pH 11, which had the largest free-space length between the SiO_2_ clusters as well as larger cluster sizes and a different structural organization to the other nanocomposites. The restrictive effect of the SiO_2_ domains in this nanocomposite on the mobility of the epoxy chains is also reflected in the increase in T_g_ (Figure 7), compared to other nanocomposites prepared with APTES.

### 4.4. The High Temperature Complex Permittivity

At higher temperatures more pronounced differences are observed between the different compositions. A common change for all the samples is the shift of the relaxations to higher frequencies with higher temperatures. This is due to the increased mobility of epoxy chains as the glass transition is approached, and movements along the chains are no longer restricted—the O-H dipoles can then keep up with the faster switching of the electrical field at higher frequencies. Although not visible in the range of frequencies measured, it is suspected that above the glass transition the β-relaxation will be entirely absent and the movement of the molecular segments will be the primary dielectric relaxation. This phenomenon is known as the α-relaxation, which is observed above 80 °C in pure epoxy at lower frequencies. With increasing temperatures, the α-relaxation is similarly shifted to subsequently higher frequencies as the molecular segments will find it easier to orient themselves with the electrical field. 

The rapid increase in real permittivity at temperatures beyond the glass transition region is accompanied by a similar increase in the imaginary permittivity (Figure 10). The slope of −0.9 to −1 for ε’’ indicates that the increase in ε’’ can be due to charge transport [39]. However, this is unlike DC conductivity (involving free-charge carriers moving continuously through the material) where ε’ would show no frequency dependence, and does not lead to charge storage [40]. The simultaneous increase in ε’ and ε’’ is instead reminiscent of the low-frequency dispersion (LFD) effect, where a strong increase in the susceptibility χ’ (and therefore the permittivity ε’) at low frequencies implies a finite and reversible storage of charge at interfaces [41]. The LFD effect occurs in carrier dominated systems and may appear similar to DC conduction—therefore it is sometimes referred to as quasi-DC (QDC) [42]. The origin of the LFD in this case is possibly due to an electrode polarization (EP) effect caused by ion blockage at the electrode-sample interface. The ions responsible for this are the residual Na^+^ and Cl^−^ ions from the synthesis of DGEBA, which accumulate at the electrodes at high temperatures [39]—this is enabled by the increased ion mobility once the epoxy becomes rubbery above the glass transition. 

**Figure 14:**
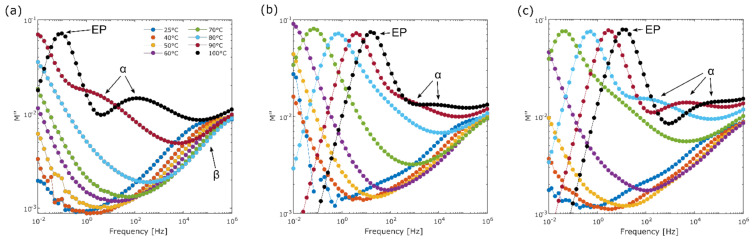
Imaginary moduli of (**a**) Pure epoxy, and nanocomposites with 5 wt.% SiO_2_ prepared at (**b**) pH 7 and (**c**) pH 11, from 25–100 °C, showing the α and β relaxations as well as the electrode polarization (EP) effect.

One problem that arises with this electrode polarization is that it is difficult to isolate the α-relaxation from the real and imaginary permittivities, especially since the corresponding peak in the imaginary permittivity is often obscured by the increase in ε’’ at low frequencies. This necessitates the use of the complex moduli instead of the permittivity, as the relaxations move to a higher frequency in the modulus spectra compared to the permittivity spectra [43], and so are less obscured by the electrode polarization (which also manifests as peaks in the imaginary moduli, *M*’’ instead of an increase in ε’’). Figure 14 shows the evolution of the α-relaxation in in *M’’* for pure epoxy and different nanocomposites (5 wt.% SiO_2_ prepared at pH 7 and 11). The α- and β-relaxations show the same trend observed in the permittivity, with shifts to higher frequencies at higher temperatures, and the α-relaxation appearing at lower temperatures in the nanocomposites. This is consistent with the changes in the glass transition behavior observed in nanocomposites prepared with APTES (Figure 7), which is also attributed to the increased mobility of the polymeric mass-fractal structures of SiO_2_, especially at low SiO_2_ content [24].

**Figure 15:**
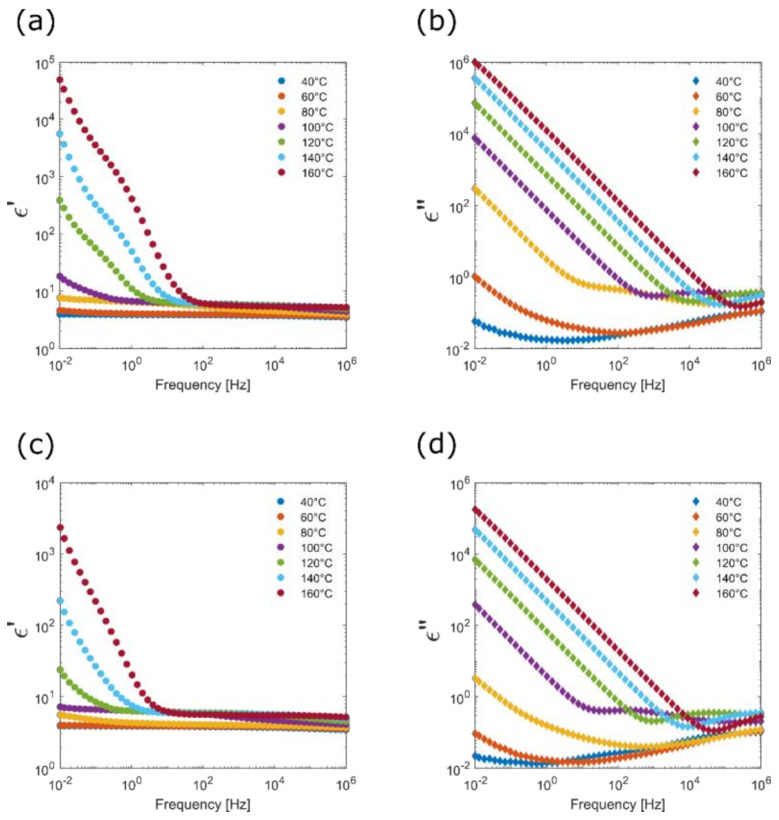
Comparison of the complex permittivities of epoxy-SiO_2_ nanocomposite (5 wt.%, pH 11) with APTES:DGEBA mass ratios of (**a**,**b**) 1:10 and (**c**,**d**) 1:30. (**a**,**c**) display the real relative permittivities, while (**b**,**d**) and display the imaginary permittivities.

The electrode polarization (EP), and the corresponding increases in ε’ and ε’’, is more significant in the nanocomposites than in pure epoxy. The peak for EP in *M*’’ in the nanocomposites is shifted to higher frequencies at the same temperature than the corresponding peak in pure epoxy—in other words, the EP appears at much lower T in the nanocomposites than in the pure epoxy. Additionally, the increase in ε’’ is between one and two orders of magnitude larger in the nanocomposites than the pure epoxy for a given temperature. This is most likely due to an increased number of charge carriers, such as ions, from the precursors used for the in situ synthesis of the nanoparticles. Yang et al. [44] have demonstrated similar behavior in ε’’ at temperatures above 100 °C when pre-synthesized SiO_2_ (modified with APTES and hyperbranched polyesters) was used in the nanocomposites. However, in that work, at lower temperatures the SiO_2_ was observed to inhibit the EP/LFD effect instead. Yeung and Vaughan [13] also reported increasing values for ε’ and ε’’ above the glass transition for epoxy nanocomposites with pre-synthesized SiO_2_ functionalized with GPTMS, although in their work the nanocomposites exhibited a more prominent EP/LFD effect. They attributed this partly to adsorbed water molecules at the interface between the epoxy and the SiO_2_, and the effect was seen to diminish with increasing amount of GPTMS used for the surface modification. However, in this work the opposite effect is observed. Figure 15 shows that the EP/LFD effect is less prominent in nanocomposites prepared with less APTES (APTES:DGEBA ratio of 1:30 instead of 1:10) at all temperatures, with lower values in both ε’ and ε’’ at 10^−2^ Hz. This implies that the EP/LFD effect is not caused by adsorbed water on the surfaces in this case (as suggested by Yeung and Vaughan), as the presence of fewer APTES molecules would mean more available sites for water to attach at the interfaces—this should amplify the increase in permittivity at high temperatures. Since the opposite is observed with a reduction in the amount of APTES, the corresponding decrease in the high temperature complex permittivity must instead be related to the changes in the structure and dispersion of the SiO_2_ domains. It is possible that the increased interconnectivity of the SiO_2_ clusters (for APTES:DGEBA of 1:10) facilitates charge transfer in the epoxy, resulting in the enhanced EP; whereas when the APTES is reduced, the increased distances between the clusters and the more limited connectivity of the network means that the charge transfer is less amplified, thereby limiting the increase in EP.

From the TEM and SAXS measurements, it is known that the structure, morphology, and dispersion of the SiO_2_ in the nanocomposites change most significantly when the amount or type of SCA is altered, and this is also reflected in the dielectric properties. The state of dispersion of nanoparticle fillers has been highlighted as a key factor in the dielectric properties of nanocomposites [5], and agglomeration is observed to generally lead to increased permittivity and dielectric losses. The results in this work highlight the importance of the structure and morphology of the inorganic filler, in addition to the quality of dispersion, to the final properties of the material. In addition, the use of the in situ route may introduce unwanted ions from the reactants that contribute to the EP/LFD, which may be detrimental to the performance of the materials at higher temperatures. 

## 5. Conclusions

The use of the sol–gel method to prepare surface-functionalized SiO_2_ in epoxy is a promising alternate route in the preparation of nanocomposites with well-dispersed nanoparticles. The selection of the amount and type of coupling agent is critical, as seen by the differences in the state of dispersion between nanocomposites prepared with APTES and GPTMS, and with different amounts of APTES. The results from the dielectric spectroscopy indicate that the structure and morphology of the inorganic components of the hybrid material are quite important: the formation of polymeric structures with mass-fractal features (from faster hydrolysis at lower pH) are more likely to contribute to the mobility of the polymer chains, thereby increasing the permittivity. The formation of more compact, cross-linked SiO_2_ domains with surface-fractal features (from faster condensation at higher pH), which more closely resembles particles with a defined shape, are more likely to inhibit the motions of the polymer chains instead, thereby reducing the permittivity. Therefore, the nanocomposites prepared at pH 11 with an APTES:DGEBA mass ratio of 1:30 exhibited the most significant reduction in the real relative permittivity (by 5% at room temperature) and in the dielectric loss tangent above 10^3^ Hz, compared to pure epoxy, which is a promising development for the use of these nanocomposites as high-voltage insulation. The next step towards such application would naturally be to investigate how the use of an in situ synthesis procedure that results in an improved dispersion of the nanoparticles, will affect the dielectric breakdown properties of the nanocomposites, in particular the inception and growth of electrical trees (pre-breakdown mechanism).

## Figures and Tables

**Table 1:** Compositions of the epoxy-SiO_2_ nanocomposites prepared via the in situ sol–gel method.

Filler Content	pH	SCA:DGEBA Mass Ratio	SCA
1–5 wt%	2 ^1^	1:10	APTES
1, 2, 3, and 5 wt%	7		
1,2 and 5 wt%	11		
5 wt%	11	1:30	
2, 3, 4, and 5 wt%	2	1:10	GPTMS

^1^ Prepared previously in [24].

**Table 2:** Structural parameters (radius of gyration R_g_) obtained from fitting the SAXS data to the unified exponential/power-law model with two structural levels. The inorganic domain size *d* was calculated using Equation (4). The fractal dimension *D* was measured from the linear slopes of the plots in Figure 4.

Sample		Structural Level	*D*	R_g_ (nm)	d (nm)
pH	Filler Content (wt.%)				
7	1 ^1^	1	1.3	-	-
	2	1	1.5	3.3 ± 0.1	8.5
		2	2.2	11.6 ± 0.9	30.0
	3	1	1.6	4.1 ± 0.4	10.6
		2	2.4	12.1 ± 1.0	31.2
	5	1	1.9	3.3 ± 0.1	8.5
		2	3.1	13.3 ± 1.7	34.3
11	1 ^1^	1	1.5	-	-
	2 ^1^	1	2	-	-
	5	1	1.9	3.3 ± 0.1	8.5
		2	3.3	14.7 ± 1.8	38.0
	5 ^1,2^	1	1.2	-	-
		2	3.5	-	-

^1^ No suitable fit was obtained with the unified model. ^2^ Reduced amount of APTES (APTES:DGEBA = 1:30).

## Data Availability

Data available upon request from the authors.

## Supplementary Materials

The following are available online at https://www.mdpi.com/article/10.3390/polym13091469/s1, Figure S1: Simultaneously acquired HAADF-STEM image and EDS maps of the nanocomposites, showing (a) the STEM image of the area mapped, and elemental maps for (b) silicon and (c) oxygen, Figure S2: (a) Procedure for processing of images for quantitative analysis and determination of the mean free-space length (L_f_). (b) Histograms produced from the computation of *L*_f_, showing the occurrences of a specific number of particle pixels found in each randomly placed box. The first histogram on the left was produced for the value of L_f_ computed automatically, while the middle and last histograms were produced from manually setting an undersized and oversized L_f_, respectively, Table S1: Values of L_f_ computed from the TEM images for the different epoxy-SiO_2_ nanocomposite samples, as well as the mean L_f_ values calculated from the computed values. The computation was run thrice for each image using 10 000 random boxes for each iteration of L_f_.
