# Supplementary material for: The Structure, Morphology, and Complex Permittivity of Epoxy Nanodielectrics with In Situ Synthesized Surface-Functionalized SiO2
Source: Polymers (Basel). 2021 May 1;13(9):1469. doi: 10.3390/polym13091469 (PMC8124689; doi:10.3390/polym13091469)
Supplement: Supplementary file 1 [file polymers-13-01469-s001.zip › polymers-1194668-supplementary.pdf]

Support Information of

The Structure, Morphology, and Complex Permittivity of Epoxy Nanodielectrics with in Situ Synthesized Surface-Functionalized SiO_2

Mohammed Mostafa Adnan [1], Inger-Emma Nylund [1], Aleksander Jaworski [2], Sverre Hvidsten [3], Marit-Helen Glomm Ese [3], Julia Glaum [1], and Mari-Ann Einarsrud [1,*]

Figure S1 shows the compositional maps obtained from energy-dispersive X-ray spectroscopy that were used to verify that the structures observed in the TEM were SiO_2. The EDS spectra were obtained using an Oxford X-Max 80 SDD detector attached to the TEM instrument, operated at a voltage of 200 kV. The data analysis was performed using the software Aztec, and the automatic element detection was set to search for Si, O, C, and N and exclude all other elements. The mapping of Si overlaps quite well with the particle clusters observed in the TEM image, whereas the O map shows that oxygen is present all over (as expected due to its presence in the DGEBA chains) but is brighter in the areas corresponding to the larger SiO_2 agglomerates.

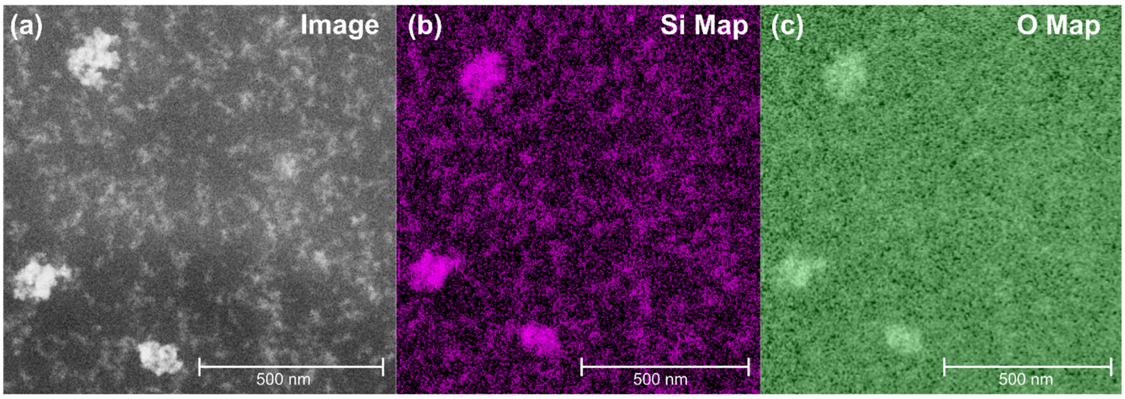

Figure S1. Simultaneously acquired HAADF-STEM image and EDS maps of the nanocomposites, showing **(a)** the STEM image of the area mapped, and elemental maps for **(b)** silicon and **(c)** oxygen.

The free-space length (L_f) was used as a quantitative measure of the state of dispersion of the nanoparticles and was calculated using binary images obtained from the TEM and STEM images. These images were first processed on the software ImageJ 1.52a using a fast-Fourier transform (FFT) bandpass filter, where large structures were filtered down to 150 pixels and small structures were filtered up to 10 pixels. This led to reduction in the noise in the background of the TEM and STEM images, allowing more accurate binary images to be prepared by thresholding, as seen in Figure S2(a). At least two (and up to four) images from different grid locations for each sample were used to check reproducibility of the results. Starting estimates of 150 nm or 300 nm for L_f in samples with APTES:DGEBA ratios of 1:10 and 1:30 were used,

respectively, and 10 000 random boxes were used to calculate L_f via iteration from the starting values.

The computations were performed on MATLAB using the code and algorithm provided by Khare and Burris [23]. On each image the computation was repeated twice for statistical significance. Table S1 shows a summary of the results from each computation, which were used in Figure 3(a). The histograms obtained from the computation display the total number of occurrences for a certain number of particle pixels being present in each of the randomly placed boxes. Figure S2(b) displays some representative histograms for (from left to right) boxes with the value of L_f calculated by the program, for a manually set undersized box, and for a manually set oversized box. If the size of the box is larger than L_f, then the likelihood of a randomly placed box containing no particles (or any particle pixel) is very small, resulting in a histogram resembling the normal distribution, and with 0 instances of a box containing no particle pixels. On the other hand, if the box is smaller than L_f, most of the randomly placed boxes will contain no particle pixels, resulting in a very skewed distribution to the left, and a low number of occurrences for boxes containing particle pixels. The algorithm for the MATLAB code attempts to find the largest box size (and therefore L_f) which gives a mode of 0 for the number of particle pixels in a box (histogram on the left in Figure S2(b)).

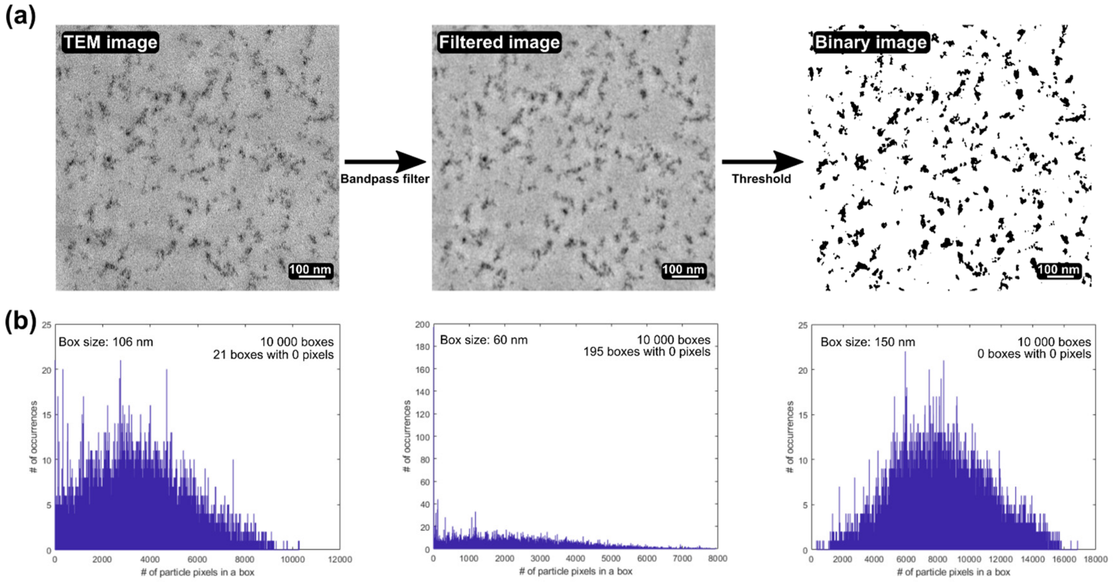

Figure S2. (a) Procedure for processing of images for quantitative analysis and determination of the mean free-space length (L_f). **(b)** Histograms produced from the computation of L_f, showing the occurrences of a specific number of particle pixels found in each randomly placed box. The first histogram on the left was produced for the value of L_f computed automatically, while the middle and last histograms were produced from manually setting an undersized and oversized L_f, respectively.

Table S1. Values of L_f computed from the TEM images for the different epoxy-SiO_2 nanocomposite samples, as well as the mean L_f values calculated from the computed values. The computation was run thrice for each image using 10 000 random boxes for each iteration of L_f.

Sample		Image	Computed values of L_f [nm]	Mean L_f [nm]
pH	Filler content [wt.%]			
2	2	1	53, 53, 53	57
		2	59, 60, 63	

	5	1	79, 81, 83	72
		2	61, 62, 65	
7	5	1	76, 79, 79	86
		2	84, 89, 91	
		3	91, 92, 93	
11	2	1	63, 63, 66	65
		2	64, 66, 67	
	5	1	106, 106, 109	105
		2	118, 119, 125	
		3	103, 106, 107	
		4	86, 88, 89	
	5 [1]	1	199, 199, 200	205
		2	206, 212, 216	

[1] Reduced amount of APTES (APTES:DGEBA = 1:30).
